# Characterization of the complete chloroplast genome of *Salvia trijuga* Diels (Lamiaceae)

**DOI:** 10.1080/23802359.2021.1991243

**Published:** 2021-10-20

**Authors:** Yan Du, Yuan-yuan Wang, Chun-lei Xiang, Mei-qing Yang

**Affiliations:** a Baotou Medical College, Baotou, Inner Mongolia, China; b Key Laboratory for Plant Diversity and Biogeography of East Asia, Kunming Institute of Botany, Chinese Academy of Sciences, Kunming Yunnan, China

**Keywords:** *Salvia trijuga*, complete chloroplast genome, phylogeny

## Abstract

The complete cp genome of *Salvia trijuga* Diels was 151,345 bp in length, including a large single-copy region (LSC) of 82,577 bp, a small single-copy region (SSC) of 17,584 bp and a pair of inverted repeats (IRs) of 25,592 bp. The genome contained 132 genes, including 87 protein-coding genes, 37 tRNA genes, and 8 rRNA genes. The overall GC content of this genome was 37.9%, with the corresponding values of LSC, SSC and IR regions being 36.0%, 31.7% and 43.1%, respectively. Further, the phylogenomic analysis strongly supported the sister relationship of *Salvia trijuga* and S*alvia plebeia* R. Br.

*Salvia trijuga* is a perennial herb of Sect. *Drymosphace* of Subgen. *Sclarea* of the genus *Salvia* L. (Lamiaceae), which is endemic to Tibet, Yunnan and Sichuan Province in China (Li and Ian 1994). It is usually locally known as “Xiao-Hong-Shen” by inhabitants of the Yunnan Province, has been occasionally used as a substitute for *Salvia miltiorrhiza* Bunge (Danshen) to treat cardiovascular diseases in folk medicine. The previous studies reported that the roots of *Salvia trijuga* contained a number of compounds, mainly Diterpenoids (Yang et al. 2003;Pan et al. 2010). In the other hand, it is confusing that *Salvia trijuga* has not formed a clade with other Dan-Shen species in phylogenetic analysis, but had closer relationships with Non-Dan-Shen species with 82% bootstrap (Wang et al. 2007). To make better use of *Salvia trijuga,* we assembled and described the complete chloroplast genome sequence of *Salvia trijuga* using the genome skimming sequencing method in this study.

Fresh leaves of *Salvia trijuga* were collected from Lijiang of Yunan, China (GPS: 100°12′53.4″N, 27°0′49.3″W). The specimen was deposited at Herbarium, Kunming Institute of Botany, CAS (KUN) (http://www.kun.ac.cn/, Xiang Chun-lei and xiangchunlei@mail.kib.ac.cn) under the voucher number D56. Total DNA was extracted under the modified CTAB method (Doyle JJ and Doyle JL 1987). The genome skimming sequencing was performed on the Illumina HiSeq 2500 platform in Novogene Bioinformatics Technology Co., Ltd. (Beijing, China). The clean reads were assembled by GetOrganelle v1.7.5.0 (Jin et al. 2020) with *Salvia miltiorrhiza* for reference (Accession number NC020431) (Qian et al. 2013). We performed annotation of the cp genome using Plann 1.1 (Huang and Cronk 2015) and manually adjusted the position of the start and stop codons. The tRNA genes were further confirmed by the online tRNAscan-SE Search Service (Lowe and Chan 2016). The complete cp genome sequence was submitted to the GenBank under the accession number MN062350. The circular map of the cp genome was drawn with the OGDRAW (Lohse et al. 2007). The genome had 132 genes comprised of 87 protein-coding genes, 37 tRNA genes, and 8 rRNA genes. Protein-coding regions accounted for 52.3% of the whole genome, whereas the tRNA and rRNA regions accounted for 1.8% and 6.0%, respectively. The overall GC content of the whole genome was 37.9%, and the corresponding values of LSC, SSC, and IR regions being 36.0%, 31.7% and 43.1%, respectively.

In order to detect the possibility of genomic data in phylogenetic analysis, all the complete cp genome sequences of *Salvia* were downloaded from GenBank and the ML phylogenetic tree with 1000 bootstraps (Maximum Likelihood) was constructed based on CDS regions with the GTR + G model in RAxML (Stamatakis 2014.) The phylogenetic results showed that the support values of most the clades in *Salvia* were 100% (Figure 1), and *Salvia trijuga* formed a sister clade with *Salvia plebeia.* Therefore, the whole chloroplast genome may be used for identifying *Saliva* species, exploiting candidate DNA markers and evaluating interspecies phylogenetic relationships.

**Figure 1.**
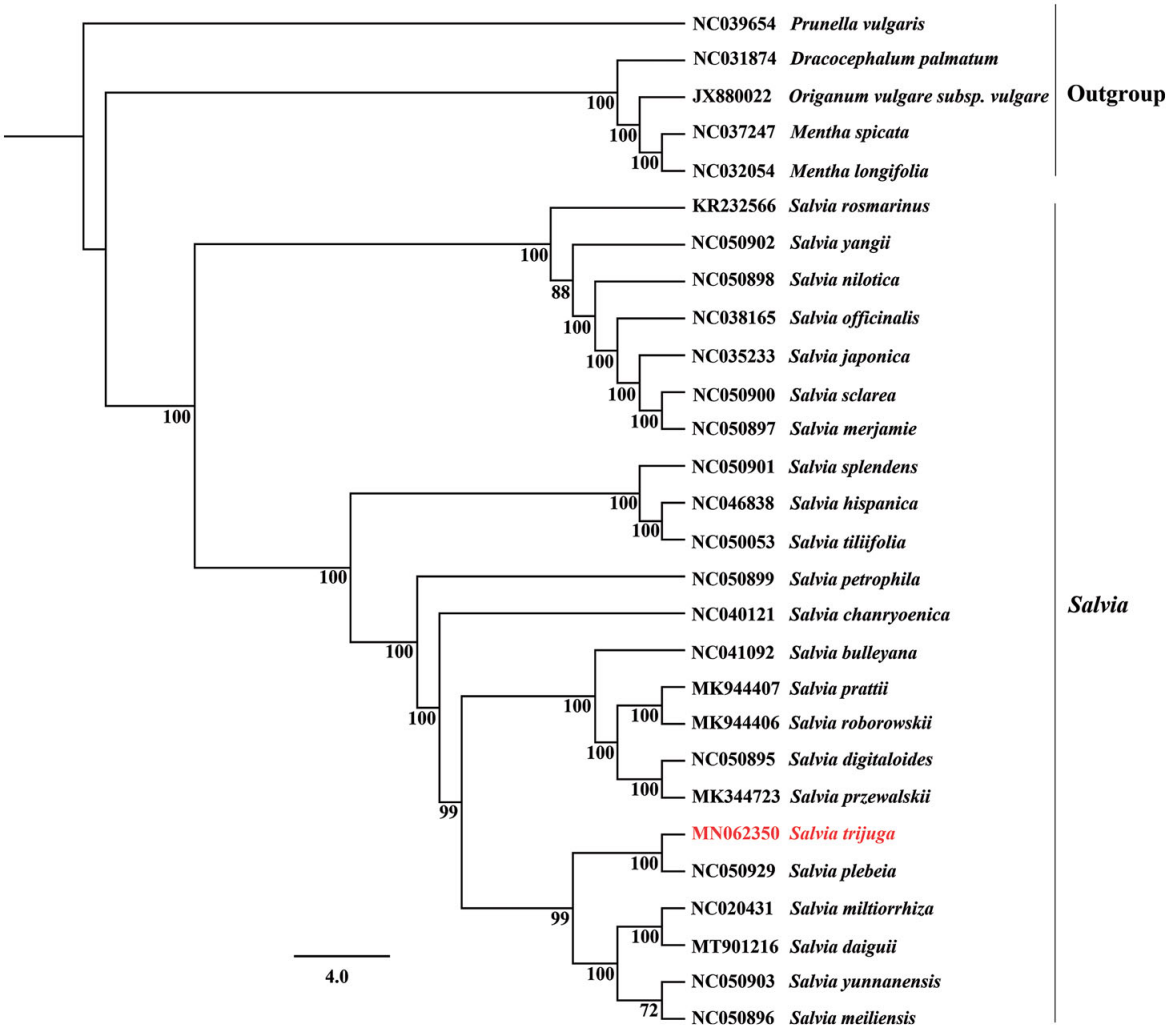
Phylogenetic tree inferred by maximum likelihood (ML) method based on CDS regions of 23 species of *Salvia* and five outgroup species, bootstrap values (%) is shown under the branch.

## Data Availability

The genome sequence data that support the findings of this study are openly available in GenBank of NCBI at (https://www.ncbi.nlm.nih.gov/) under the accession no. MN062350. The associated BioProject, SRA, and Bio-Sample numbers are PRJN743163, SRS9371381, and SAMN20000552 respectively.
